# Transcriptome analysis of flathead grey mullet (*Mugil cephalus*) ovarian development induced by recombinant gonadotropin hormones

**DOI:** 10.3389/fphys.2022.1033445

**Published:** 2022-11-01

**Authors:** Sandra Ramos-Júdez, Theodoros Danis, Nelina Angelova, Alexandros Tsakogiannis, Ignacio Giménez, Costas S. Tsigenopoulos, Neil Duncan, Tereza Manousaki

**Affiliations:** ^1^ IRTA, Sant Carles de la Ràpita, Spain; ^2^ Institute of Marine Biology, Biotechnology and Aquaculture (IMBBC), Hellenic Centre for Marine Research (H.C.M.R.), Heraklion, Greece; ^3^ Rara Avis Biotec, S.L., Valencia, Spain

**Keywords:** recombinant gonadotropin hormones, recombinant follicle-stimulating hormone, recombinant luteinizing hormone, induced vitellogenesis, ovarian RNA-seq, *Mugil cephalus*

## Abstract

**Background:** Treatment with recombinant gonadotropin hormones (rGths), follicle-stimulating hormone (rFsh) and luteinizing hormone (rLh), was shown to induce and complete vitellogenesis to finally obtain viable eggs and larvae in the flathead grey mullet (*Mugil cephalus*), a teleost arrested at early stages of gametogenesis in intensive captivity conditions. This study aimed to investigate the transcriptomic changes that occur in the ovary of females during the rGths-induced vitellogenesis.

**Methods:** Ovarian samples were collected through biopsies from the same five females at four stages of ovarian development. RNASeq libraries were constructed for all stages studied, sequenced on an Illumina HiSeq4000, and a *de novo* transcriptome was constructed. Differentially expressed genes (DEGs) were identified between stages and the functional properties of DEGs were characterized by comparison with the gene ontology and Kyoto Encyclopedia. An enrichment analysis of molecular pathways was performed.

**Results:** The *de novo* transcriptome comprised 287,089 transcripts after filtering. As vitellogenesis progressed, more genes were significantly upregulated than downregulated. The rFsh application induced ovarian development from previtellogenesis to early-to-mid-vitellogenesis with associated pathways enriched from upregulated DEGs related to ovarian steroidogenesis and reproductive development*,* cholesterol metabolism, ovarian growth and differentiation, lipid accumulation, and cell-to-cell adhesion pathways. The application of rFsh and rLh at early-to-mid-vitellogenesis induced the growth of oocytes to late-vitellogenesis and, with it, the enrichment of pathways from upregulated DEGs related to the production of energy, such as the lysosomes activity*.* The application of rLh at late-vitellogenesis induced the completion of vitellogenesis with the enrichment of pathways linked with the switch from vitellogenesis to oocyte maturation.

**Conclusion:** The DEGs and enriched molecular pathways described during the induced vitellogenesis of flathead grey mullet with rGths were typical of natural oogenesis reported for other fish species. Present results add new knowledge to the rGths action to further raise the possibility of using rGths in species that present similar reproductive disorders in aquaculture, the aquarium industry as well as the conservation of endangered species.

## Introduction

Oogenesis, *i.e.*, the development of female gametes (ova or eggs), is the process of development from oogonia, the precursor of oocytes, to mature ova that can be fertilised. In teleost, as in other vertebrates, oogenesis is controlled by the brain-pituitary-gonad axis, wherein the pituitary glycoprotein hormones, gonadotropins (Gths) play a crucial regulatory role. Two types of Gths, the follicle-stimulating hormone (Fsh) and the luteinizing hormone (Lh), are produced and released into the bloodstream by the pituitary gland under the stimulation of the hypothalamus-produced gonadotropin-releasing hormone (GnRH) (Marques et al., 2000). The Gths bind to their cognate receptors in the gonads and regulate oogenesis mainly through the stimulation of gonadal steroidogenesis, which is the ultimate mediator of the different developmental stages. In broad terms, the principal role of Fsh is to promote the onset of vitellogenesis through the stimulation of steroidogenesis in the follicular cells—hepatic synthesis of vitellogenins and uptake by the oocyte to be processed into yolk globules (Lubzens et al., 2010; Reading et al., 2017). On the other side, Lh is mostly involved in regulating the late stages of oogenesis, including oocyte maturation and ovulation (Lubzens et al., 2010).

The technology to produce recombinant Gths (rGths), recombinant Fsh (rFsh) and Lh (rLh), through the expression of their cDNAs in heterologous eukaryotic systems such as yeast, insect cells, and mammalian cell lines has helped to develop studies to determine the specific functionality of Gths (Levavi-Sivan et al., 2010; Molés et al., 2020). The recombinant Gths have also been tested as therapeutic agents to overcome problems that arrest oocyte development in several fish species (Ko et al., 2007; Kazeto et al., 2008; Giménez et al., 2015; Aizen et al., 2017; Sanchís-Benlloch et al., 2017; Palma et al., 2018; Salwany et al., 2019; Molés et al., 2020; Nguyen et al., 2020). Like many wild animals held in captivity, many fish species of high interest for aquaculture exhibit reproductive dysfunctions due to the captive environment. Female fish exhibit different failures or dysfunctions during oogenesis, mainly mediated through the endocrine system by postponing or suspending the release of Gths from the pituitary (Mylonas et al., 2010).

The flathead grey mullet (*Mugil cephalus*) is an important marine fish species in Asian and Mediterranean countries (González-Castro et al., 2016) with a worldwide fisheries capture of 101,765 tons and an aquaculture production of only 5,418 tons in 2020 (FAO, 2021). In recent decades, the flathead grey mullet has been identified as an important species for the diversification of European aquaculture, however, its culture is mainly limited due to a reproductive dysfunction this species shows when maintained in intensive conditions. Females remain arrested at previtellogenesis and do not undergo vitellogenesis (Ramos-Júdez et al., 2021) and, inevitably, aquaculture production is based mainly on the capture of wild juveniles or broodstock, which is not sustainable in the long term (Yousif et al., 2010). Therefore, artificial hormone manipulation is needed to induce the mullet previtellogenic gonad to enter into vitellogenesis, complete oocyte development to maturation and ovulation, and obtain eggs and larvae. Recently, the induction of vitellogenesis to produce fertilized eggs that hatched to produce larvae was achieved with a therapy using homologous single-chain rFsh and rLh produced in Chinese Hamster Ovary cells (Ramos-Júdez et al., 2021; Ramos-Júdez et al., 2022), whereas in comparison untreated control females remained at previtellogenic gonadal arrest (Ramos-Júdez et al., 2021). Although rGths have previously been used to induce oogenesis in other fish species (Ko et al., 2007; Kazeto et al., 2008; Giménez et al., 2015; Palma et al., 2018; Nguyen et al., 2020), no other study reported the induction and completion of vitellogenesis to obtain viable eggs. These achievements in the use of exogenously applied rGths to induce maturation, indicate that the study of stage-specific molecular variations underlying the ovarian development under the stimulation by rGths might provide insights into their direct role in the flathead grey mullet oogenesis.

To date, large-scale studies for the transcriptome of the teleost ovary have improved the knowledge of the molecular and cellular mechanisms of this complex process in fish by examining the transcriptomic signalling during the reproductive cycle (Goetz et al., 2006; Santos et al., 2007; Cerdà et al., 2008; Luckenbach et al., 2008; Bobe et al., 2009; Tingaud-Sequeira et al., 2009; Villeneuve et al., 2010; Breton et al., 2012; Kleppe et al., 2014). Moreover, recent studies applying bulk RNA-sequencing (RNA-Seq), an accurate and sensitive technique (Wang et al., 2009), have elucidated the mechanisms of ovary differentiation (Cai et al., 2017; Han et al., 2019) and transcriptomic changes during different stages of ovarian development in a natural breeding season in non-model organisms (Reading et al., 2012; Martyniuk et al., 2013; Gioacchini et al., 2019).

In this context, this work aimed to unravel the transcriptomic changes that occur in the ovary of flathead grey mullet females during rGth-induced vitellogenesis. We used an RNA-Seq approach and *de novo* transcriptome assembly of samples collected at previtellogenic arrested stage and at different time points during the ovarian development under the stimulation of rGths as described in the study conducted by Ramos-Júdez et al. (2021). These data are expected to constitute a resource for elucidating the molecular mechanisms that underlie ovarian development induced by rGths which will improve the development of induction protocols and facilitate the breeding of flathead grey mullet and other species with similar reproductive dysfunctions.

## Material and methods

### Animals and experimental design

**FIGURE 1:**
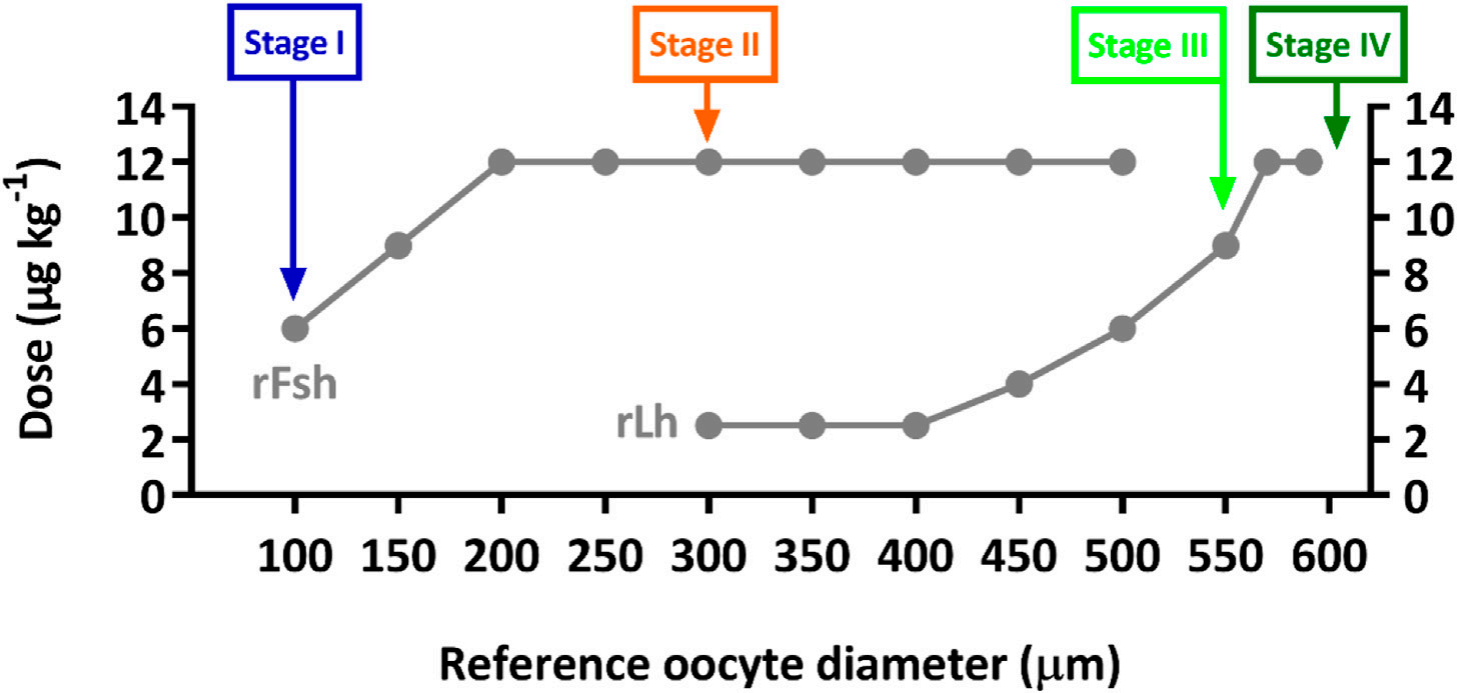
Graphical representation of the recombinant gonadotropin hormonal treatment from Ramos-Júdez et al. (2021), identifying the points at which ovarian samples were collected by cannulation from five flathead grey mullet females. Stage I, before rGth treatment from initial arrested gonad; Stage II, after 4 weeks of increasing doses of rFsh (from 6, 9–12 μg kg^−1^); Stage III, after combined treatment with 12 μg kg^−1^ rFsh and increasing doses of rLh (2.5, 4–6 μg kg^−1^); and Stage IV, after rLh treatment (doses of 9 and 12 μg kg^−1^) administered on a three-day-basis until completion of oocyte growth to ∼600 µm.

Five flathead grey mullet (*Mugil cephalus*) females (mean ± SD, 915 ± 126 g initial body weight; 38 ± 3 cm standard length) that were arrested at early stages of gonad development (previtellogenesis) were selected from an experimental group and were sampled to follow ovarian development induced with species-specific rGths as described in Ramos-Júdez et al. (2021). Briefly, the fish were weekly treated with increasing doses of rFsh—from 6, 9–12 μg kg^−1^ — and after 4 weeks, rFsh was combined with rLh—at increasing doses of 2.5, 4–6 μg kg^−1^—. Then, when oocyte diameter was ≥550 μm, only rLh —9 and 12 μg kg^−1^— was administered on a three-day-basis to complete oocyte growth to ∼600 µm (Figure 1). Broodstock origin and culture conditions are further detailed in Ramos-Júdez et al. (2021).

Ovarian samples were collected by cannulation from the same five females at four sampling points during the induced gonadal development 1) before rGth treatment from the initial arrested gonad (Stage I, Day 0), 2) after four weeks of rFsh administration (Stage II, Day 28), 3) after the combined treatment with rFsh and rLh (Stage III, Day 63), and 4) after rLh treatment (Stage IV, Day 6–12 after achieving ≥ 550 µm) (Figure 1). At each sampling procedure, fish were first placed in a tank with 73 mg L^−1^ of MS-222 (Sigma-Aldrich, Spain) and moved to a recipient with 65 mg L^−1^ of MS-222 for manipulation. Fish were checked until complete recovery from anaesthesia as well as periodically observed to detect any abnormal behaviour. Water parameters were regularly checked and controlled to maintain high levels of water quality and animal welfare.

### Ovarian biopsy collection

Biopsy samples were aspirated into a plastic cannula inserted through the urogenital pore and were divided into three portions. One portion was expelled into 2 ml Eppendorf tubes containing RNAlater^®^ (Sigma-Aldrich, Spain) held at 4°C before storage at −80°C until further processing. The second portion was used for *in situ* measurement of the diameter of the largest oocytes (n = 20) by light microscopy (Zeiss Axiostar Microscope) and the third portion was fixed in Bouin’s solution for 24 h and storage in 70% ethanol for histological analysis.

### Histological analysis

Ovarian samples fixed in Bouin’s solution and kept at 70% ethanol, were dehydrated in ascending grades of ethanol, embedded in paraffin, then sectioned at 3 μm thickness and stained with hematoxylin and eosin (Casa Álvarez, Spain). The histological slides were observed under a light microscope (Leica DMLB, Houston, United States). The percentages of different stages of oocyte development were calculated by counting 100–150 oocytes in each gonad sample. The maturation stage of females was determined according to the most developed and abundant stage of oocytes present.

### RNA extraction, library preparation and sequencing

Total RNA was extracted from the twenty ovarian samples (5 females × 4 ovarian stages) using the RNeasy^®^ extraction kit (Qiagen) following the manufacturer’s recommendations that include an on-column DNase digestion to remove gDNA from total RNA preparations. The amount of isolated RNA was measured by spectrophotometry (NanoDrop^®^ ND-2000, Thermo Scientific™) and its integrity was assessed through agarose gel electrophoresis (2%) according to Masek et al. (2005). Transcriptome libraries were constructed from the five biological replicates for each state (Stage I-1/2/3/4/5, Stage II-1/2/3/4/5, Stage III-1/2/3/4/5 and Stage IV-1/2/3/4/5). Libraries were prepared with the Illumina TruSeqTM RNA Sample Preparation Kits following the manufacturer’s protocol and sequenced as 150 bp paired-end reads in a single Illumina HiSeq 4,000 lane performed at the Norwegian Sequencing Centre (Oslo, Norway).

### *De novo* transcriptome assembly

Raw reads quality control was performed using FastQC v0.11.8 (http://www.bioinformatics.babraham.ac.uk/projects/fastqc/) and reads were pre-processed using Trimmomatic v0.39 (Bolger et al., 2014) to remove those containing adapters and low-quality reads, with parameters set to “ILLUMINACLIP:TruSeq3-PE.fa:2:30:10:2:keepBothReadsSLIDINGWINDOW:4:15 LEADING:10 TRAILING:10 MINLEN:75 AVGQUAL:30”. Then, all samples’ trimmed reads were assembled together to obtain a single transcriptome. Trinity software v2.8.5 was used for *de novo* assembly with default parameters settings (Kmer = 25) (Grabherr et al., 2013; Haas et al., 2013). Assembled transcriptome completeness was assessed with BUSCO v3.1.0 (Simão et al., 2015) using the vertebrate orthologs database as reference. Trimmed reads of each gonad sample were mapped back separately against the assembly with Bowtie2 (Langmead and Salzberg, 2012) and the calculation of relative abundances was performed by RSEM through a Trinity script (Li and Dewey, 2011). Gene expression given as Fragments Per Kilobase of transcript per Million mapped read (FPKM) was calculated and transcripts with less than 1 FPKM were excluded. All computations were performed at the high-performance computing bioinformatics platform of HCMR (Crete, Greece) (Zafeiropoulos et al., 2021a).

### Transcriptome functional annotation

The assembled transcripts were functionally annotated using Trinotate pipeline v3.2.1 with *e*-value cut-off of 10^–5^ (https://trinotate.github.io/). TransDecoder v5.5.0 (http://transdecoder.github.io) was run to predict coding peptide sequences within the transcripts and transform the longest open reading frame (ORF) of 100 codons or more into peptide sequences. The sequence similarity of the assembled transcripts with more than 1 FPKM was evaluated using BLASTX (NCBI-blast 2.9.0+) against diverse databases (UniProtKB/SwissProt database, Kyoto Encyclopedia of Genes and Genomes (KEGG) and Gene Ontology (GO)). The TransDecoder peptide sequence file for final candidate longest-ORF was searched for amino acid sequence homologies using BLASTP (NCBI-blast 2.9.0+, *e*-value cut-off of 10^–5^). In order to identify conserved protein families among the predicted peptide sequences, HMMER hmmscan (v3.3) (Finn et al., 2011) was used for protein domain identification against Protein family (Pfam) database. In addition, SignalP (v4.1) (Petersen et al., 2011) was used to predict the presence of signal peptides, and the TMHMM (v2.0) (Krogh et al., 2001) was used to predict transmembrane helices within the predicted ORFs. All the outputs from BLASTX, BLASTP, HMMER, THMM and SignalP were loaded into the Trinotate SQLite database and generated a flat file report containing all annotation information for each contig.

### Quantification and analysis of differentially expressed genes

Count data matrix from the filtered transcriptome was constructed and imported in R v3.6.1 (R Core Team. R, 2021). Genes with less than thirty reads in all samples were excluded prior to differential expression analysis to improve the statistical power. To visually explore the global gene expression pattern in the samples, a principal component analysis (PCA) was performed on the normalized counts after the variance stabilizing transformation (VST) (Benjamini and Hochberg, 1995; Love et al., 2014) (including all the genes which sum of counts in each row >30). Differential expression analysis was performed on raw reads by DESeq2 v1.26.0 under Bioconductor package, which uses the Benjamini and Hochberg (1995) method for multiple testing correction of the *p*-values obtained by the Wald test. Pairwise differential expression analyses were performed between gonadal stages (Stage I vs. II, Stage II vs. III, Stage III vs. IV, Stage I vs. III and Stage I vs. IV) with special attention to comparison of gonadal stages in time lap sequence (Stage I vs. II, Stage II vs. III, Stage III vs. IV). Genes with an adjusted *p*-value < 0.05 were considered to show statistically significant differential expression.

To obtain the DEGs that were specifically expressed or shared between determinate stages, up/downregulated Venn diagrams were produced first for the DEGs obtained from the comparisons in time-lap sequence (Stage I vs. II, Stage II vs. III, and Stage III v IV), and second for the DEGs obtained from the comparisons of Stage I with II, III and IV.

### Enrichment analysis of DEGs

To gain an insight into the biological roles of the significant up- or downregulated genes in ovarian stages in time-lap sequence (Stage I vs. II, Stage II vs. III, Stage III vs. IV), GO and KEGG pathway enrichment analysis were performed. For this, the enrichment factor was calculated as the number of differentially expressed genes in a specific GO term or KEGG pathway divided by the total number of genes in this GO term or KEGG pathway. The statistic of enrichment, which is the probability of observing *k* or more significantly expressed genes in the pathway by chance, was calculated as the sum of the mass functions—following a hypergeometric equation—for each gene count that is greater than or equal to the number of genes observed (Karp et al., 2021). GO terms and KEGG pathways of DEGs with *p*-value < 0.05 were considered to show statistically significant enrichment.

## Results

### Stages of ovarian development

To identify differentially expressed genes during ovarian development induced by rGths in *Mugil cephalus*, samples of ovarian tissue were obtained throughout the treatment. The histological sections showed that the flathead grey mullet presented a group-synchronous ovary; there was one abundant cluster of oocytes recruited into vitellogenesis that advanced synchronously through the different stages of this process.

**FIGURE 2:**
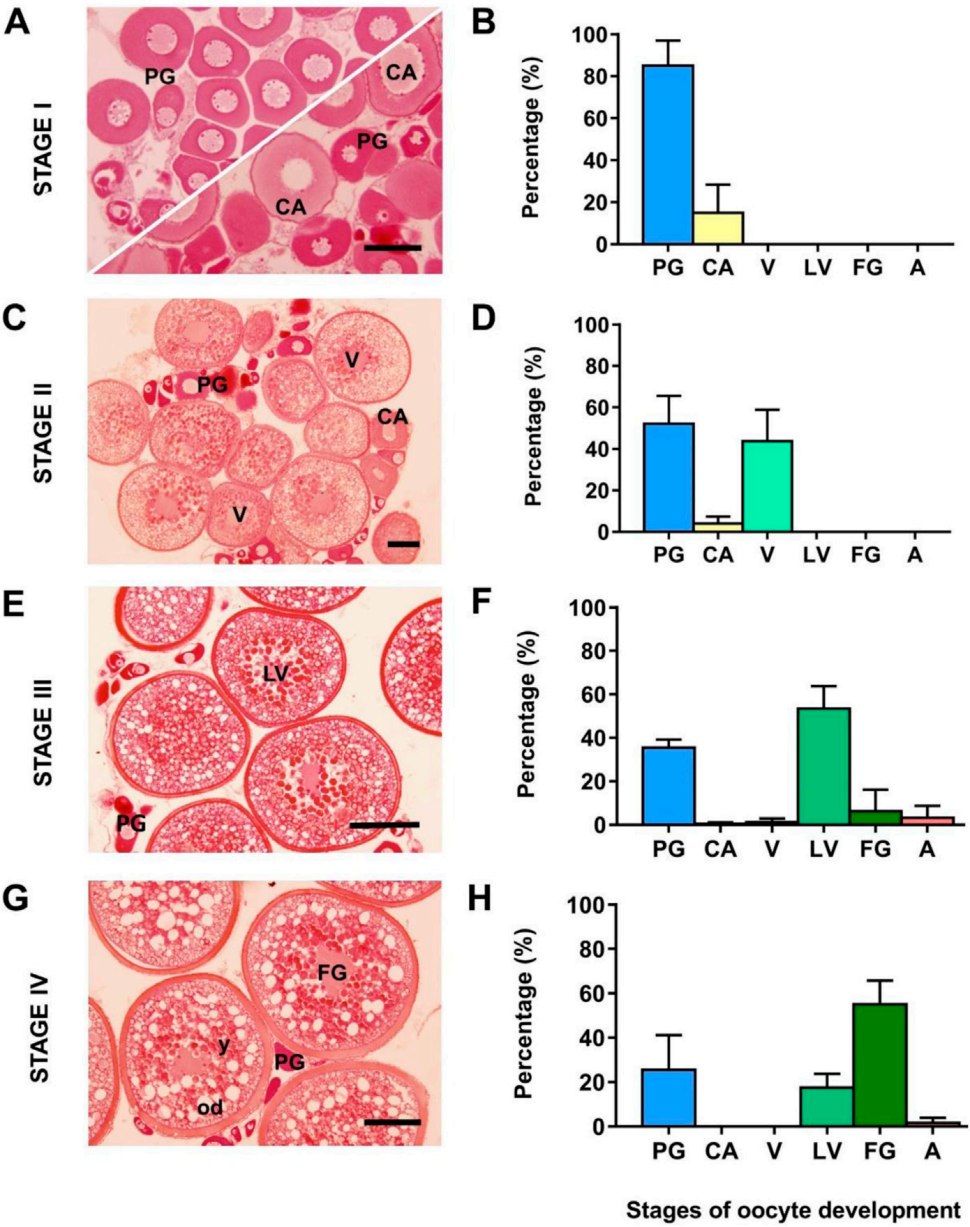
Development of the flathead grey mullet (*Mugil cephalus*) ovaries representing the four points at which samples used for transcriptome sequencing were collected. Light micrographs **(A,C,E and G)** of oocytes stained with hematoxylin-eosin are representative of the fish used. Frequency of oocytes in the ovary at each sampling point **(B,D,F and H)** at different developmental stages are presented as the mean ± SD (n = 5 females per stage). **(A)** Previtellogenic ovary **(C)** early-to-mid vitellogenic ovary **(E)** late-vitellogenic ovary; and **(G)** ovary with full-grown oocytes. PG, primary growth oocytes; CA, cortical alveolus stage; V, early-to-mid-vitellogenesis; LV, late-vitellogenesis; FG, full-grown oocyte; od, oil droplets; y, yolk. Scale bars correspond to 100 μm **(A and C)** and 200 μm **(E and G)**.

Females before hormone treatment (Stage I) were arrested at previtellogenesis (before the appearance of yolky oocytes); three females had all oocytes at primary growth and two females presented some oocytes that had initiated the secondary growth phase with the accumulation of cortical alveoli around the periphery of the oocyte and inward to the nucleus (mean diameter of the most developed oocytes: 100 ± 17 µm) (Figures 2A,B). Samples collected after rFsh treatment (Stage II) were undergoing vitellogenesis and presented, together with previtellogenic oocytes, an abundant clutch of yolky oocytes at early-to-mid-vitellogenesis with a maximum diameter of 323 ± 46 µm (Figures 2C,D). Samples obtained after combined treatment of rFsh and rLh (Stage III) mostly presented oocytes at late-vitellogenesis with a maximum diameter of 559 ± 15 µm (Figures 2E,F), whereas samples collected after rLh (Stage IV) mainly presented full-grown vitellogenic oocytes with a maximum diameter of 603 ± 3 µm (Figures 2G,H).

### Quality control, trimming and *de novo* assembly

Illumina HiSeq 4,000 paired-end sequencing generated a total of 614,942,156 raw paired reads (307,471,078 read pairs) of which 506,875,944 paired reads were maintained after trimming (82.43%) (Table 1). *De novo* assembly produced initially 513,643 transcripts with an average contig length of 919.18 nucleotides (nt), N50 value of 1,561 nt while the average GC content was 43.61%. BUSCO revealed an 86.4% of transcriptome completeness (96% complete + fragmented). BUSCO assessment results further indicated: 53.4% of complete and duplicated BUSCOs, 9.6% fragmented, and 4% were missing. Moreover, an average of 89.68% of the reads were successfully back-mapped on the assembled transcriptome (Table 1). Transcripts with an expression value of FPKM ≥1 were filtered and constituted the final assembly of 287,089 transcripts. This set of filtered transcripts had an average size of 798.43 nt, N50 value of 1,539 nt and an average GC content of 44.13%.

**TABLE 1:** Overview of RNA-Seq reads and mapping back to the assembled transcriptome.

Sample	Total raw paired reads	Total trimmed paired reads (ratio %)	Total mapped reads (ratio %)
Stage I - 1	35,289,446	28,688,398 (81.29)	26,396,195 (92.01)
Stage I - 2	28,957,550	22,272,550 (76.91)	20,423,928 (91.70)
Stage I - 3	26,395,762	21,502,258 (81.46)	19,644,463 (91.36)
Stage I - 4	35,329,986	29,616,570 (83.83)	26,785,226 (90.44)
Stage I - 5	31,666,710	26,290,908 (83.02)	23,974,679 (91.19)
Stage II - 1	27,951,938	23,088,548 (82.60)	21,084,462 (91.32)
Stage II - 2	27,696,758	23,183,714 (83.71)	21,055,449 (90.82)
Stage II - 3	27,949,986	23,023,802 (82.38)	20,661,560 (89.74)
Stage II - 4	29,931,924	24,872,658 (83.10)	22,353,058 (89.87)
Stage II - 5	33,041,106	27,564,656 (83.43)	24,959,796 (90.55)
Stage III - 1	32,244,488	26,676,528 (82.73)	23,971,528 (89.86)
Stage III - 2	32,054,802	26,656,800 (83.16)	23,935,141 (89.79)
Stage III - 3	36,213,184	30,209,676 (83.42)	27,028,597 (89.47)
Stage III - 4	31,790,304	26,881,922 (84.56)	24,126,525 (89.75)
Stage III - 5	28,951,286	22,595,958 (78.05)	20,367,997 (90.14)
Stage IV - 1	27,270,110	22,687,576 (83.20)	19,935,573 (87.87)
Stage IV - 2	24,525,510	20,159,616 (82.20)	17,972,298 (89.15)
Stage IV - 3	31,695,898	26,355,426 (83.15)	21,295,184 (80.80)
Stage IV - 4	32,273,266	26,954,152 (83.52)	24,169,788 (89.67)
Stage IV - 5	33,712,142	27,594,228 (81.85)	24,310,515 (88.10)
**Total**	614,942,156	506,875,944 (82.38)	454,566,347 (89.68)

### Transcriptome annotation

The SwissProt, GO and KEGG databases were employed for the annotation of the 287,089 sequences. The BLASTx against the Swiss-Prot databases resulted in 58,306 (20.3%) transcript gene assignments using 1 e^−5^ as the *e*-value cutoff. A total of 57,021 (19.9%) sequences had a match against the GO database, of which 50,268 (88.2%) representing biological processes (BC), 53,992 (94.7%) associated with cellular components (CC) and 48,190 (84.5%) matching molecular function (MF). A total of 51,237 (17.8%) sequences were associated with a KEGG pathway.

### Identification of DEGs

**FIGURE 3:**
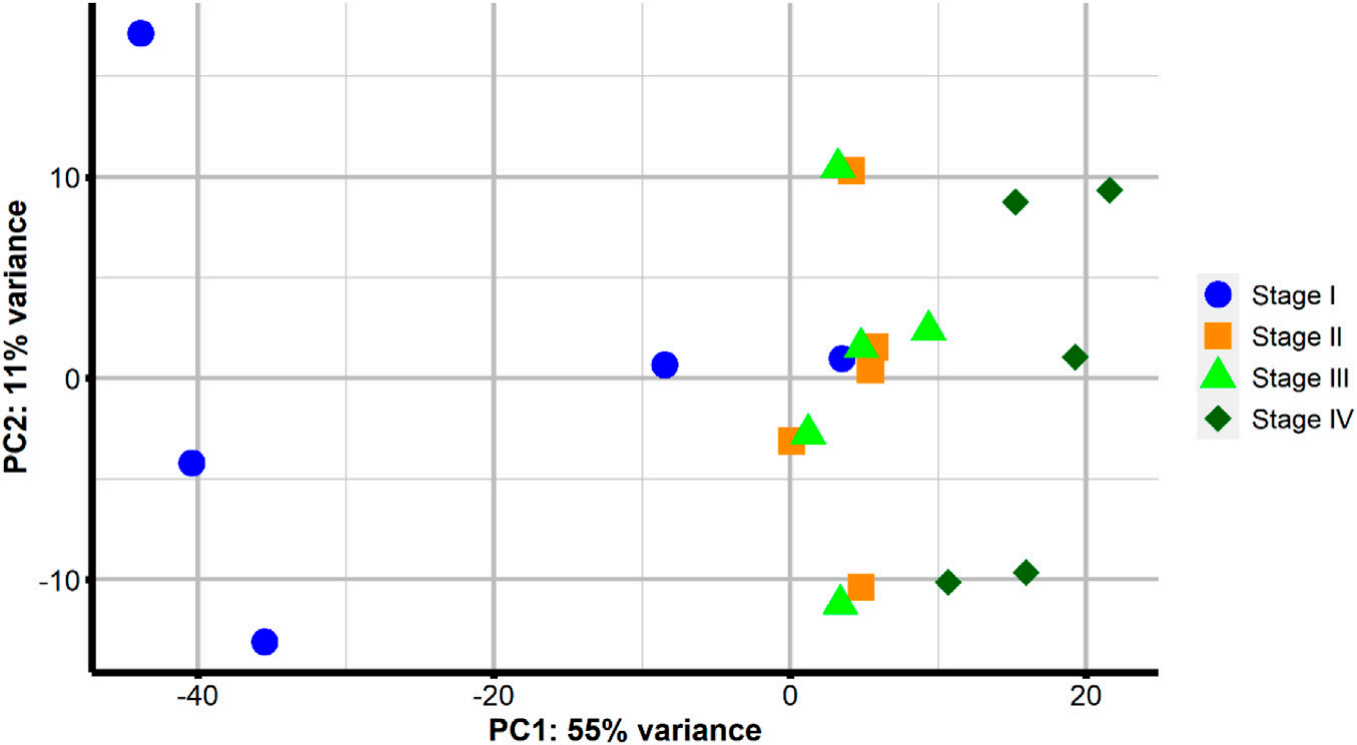
Principal component analysis of sample-to-sample Euclidean distances following VST transformation of gene counts in samples from four points of sampling: at previtellogenic arrested gonadal development before rGth treatment (Stage I–blue circle); at early-to-mid-vitellogenesis after 4 weeks of rFsh treatment (Stage II–orange square); at late-vitellogenesis obtained after the combination of rFsh and rLh (Stage III–light green triangle); and full-grown oocytes after the application of rLh to induce the latest stages of oocyte growth (Stage IV–dark green rhombus).

The PCA (Figure 3) of the Euclidean distance of the VST counts showed samples tended to group together according to the stage, with a wide scattering of some samples from Stage I. These five Stage I females were all at previtellogenesis, but three females only had primary growth oocytes (blue circle points on the left of Figure 3), while two females had some oocytes with cortical alveolus (blue circle points on the right of Figure 3). While there was an obvious separation between samples obtained at Stage I and Stage IV, samples from Stages II and III grouped together. In this general overview, component 1 of the PCA explained 55% of the variance, while component 2 contributed to 11% of the variance.

A total of 8,954, 1,113 and 1,587 DEGs were found in the comparisons of Stages I – II, II – III, and III – IV, respectively. The analysis of DEGs throughout oogenesis (Supplementary Figure S1) showed that 6,147 genes were significantly upregulated from previtellogenesis (Stage I) to early-to-mid-vitellogenesis induced with rFsh treatment (Stage II); 814 genes were upregulated from Stage II to advanced vitellogenesis (Stage III) obtained after the rFsh and rLh combined treatment, while 994 genes were upregulated in the transition from Stage III to full-grown oocytes (Stage IV) after rLh application. The corresponding numbers for downregulated genes were 2,807, 299, and 593, respectively. Throughout oogenesis, more gene transcripts were upregulated than downregulated and smaller differences in the number of DEGs were observed in consecutive stages of development or sampling points rather than in separated developmental stages. The largest difference was detected from previtellogenesis (Stage I) to full-grown oocytes (Stage IV) with 23,169 upregulated and 4,965 downregulated DEGs, and the smallest difference was observed between the vitellogenic stages II and III.

Some DEGs were only specifically expressed between two developmental stages but not between others (Supplementary Figure S2). Almost all DEGs obtained in the comparisons of consecutive stages of gonadal development (Stage I - II, Stage II - III, and Stage III - IV) were stage-specific (Supplementary Figures S2A,B). Among the DEGs obtained in the comparison of Stage I with II, III and IV, there were 164, 1,646, and 10,602 stage-specific DEGs with upregulation in Stage I vs. II, I vs. III and I vs. IV, respectively (Supplementary Figure S2C), and there were 282, 613, and 1,508 stage-specific DEGs with downregulation in Stage I to II, I to III and I to IV, respectively (Supplementary Figure S2D).

**FIGURE 4:**
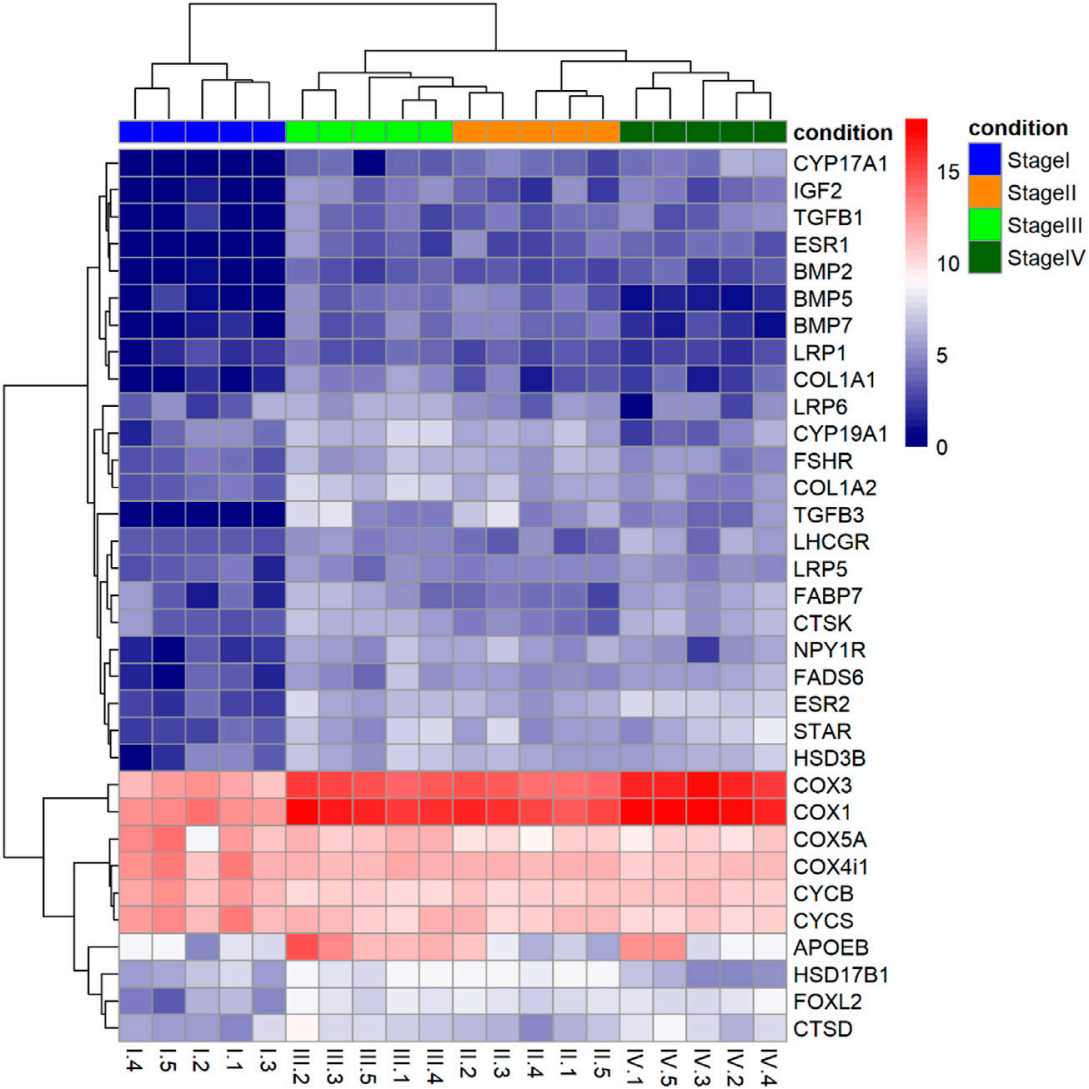
Heatmap of Log2 fold changes of a set of genes selected due to their known role in processes related to vitellogenesis such as steroidogenesis, lipid metabolism and transport, and energy production. Each column represents samples and all samples were divided to two clusters based on their stage (previtellogenesis and vitellogenesis). Hierarchical clustering for the gene expression matrix was based on the Euclidean correlation. The gradual colour from blue to red represents the expression changes of genes.

A detailed analysis of DEGs (Figure 4) identified steroidogenic-related genes that were upregulated at different stages during vitellogenesis: acute regulatory protein (*star*), the 3β-hydroxysteroid dehydrogenase (*hsd3b*), the ovarian form of Cytochrome P450 aromatase (*cyp19a1*), 17β-hydroxysteroid dehydrogenase 1 (*hsd17b1*), P450 17-Alpha-Hydroxylase/17,20 Lyase (*cyp17a1*) and estrogen receptors (*esr1* and *esr2*). Also, a set of genes related to the insulin-like growth factor (IGF) system, the transforming growth factor β family (TGB-β), and the bone morphogenic proteins family (BMP) of growth factors were upregulated during vitellogenesis. The pattern of expression of other genes was also identified, among them; genes related to lipid metabolism (*fads6*) and transport (*fapd7, apoeb, lrp1, lrp5, lrp6*), to the electron transport chain and oxidative phosphorylation (*i.e., cox1, cox3, cycs, cycb*), cytoskeletal-related transcripts such as collagen (*col1a1, col1a2*), and lysosomal cathepsins (*ctsd, ctsk*), or the gonadotropin hormones receptors (*fshr* and *lhcgr*).

### Gene ontology functional analysis of DEGs

**FIGURE 5:**
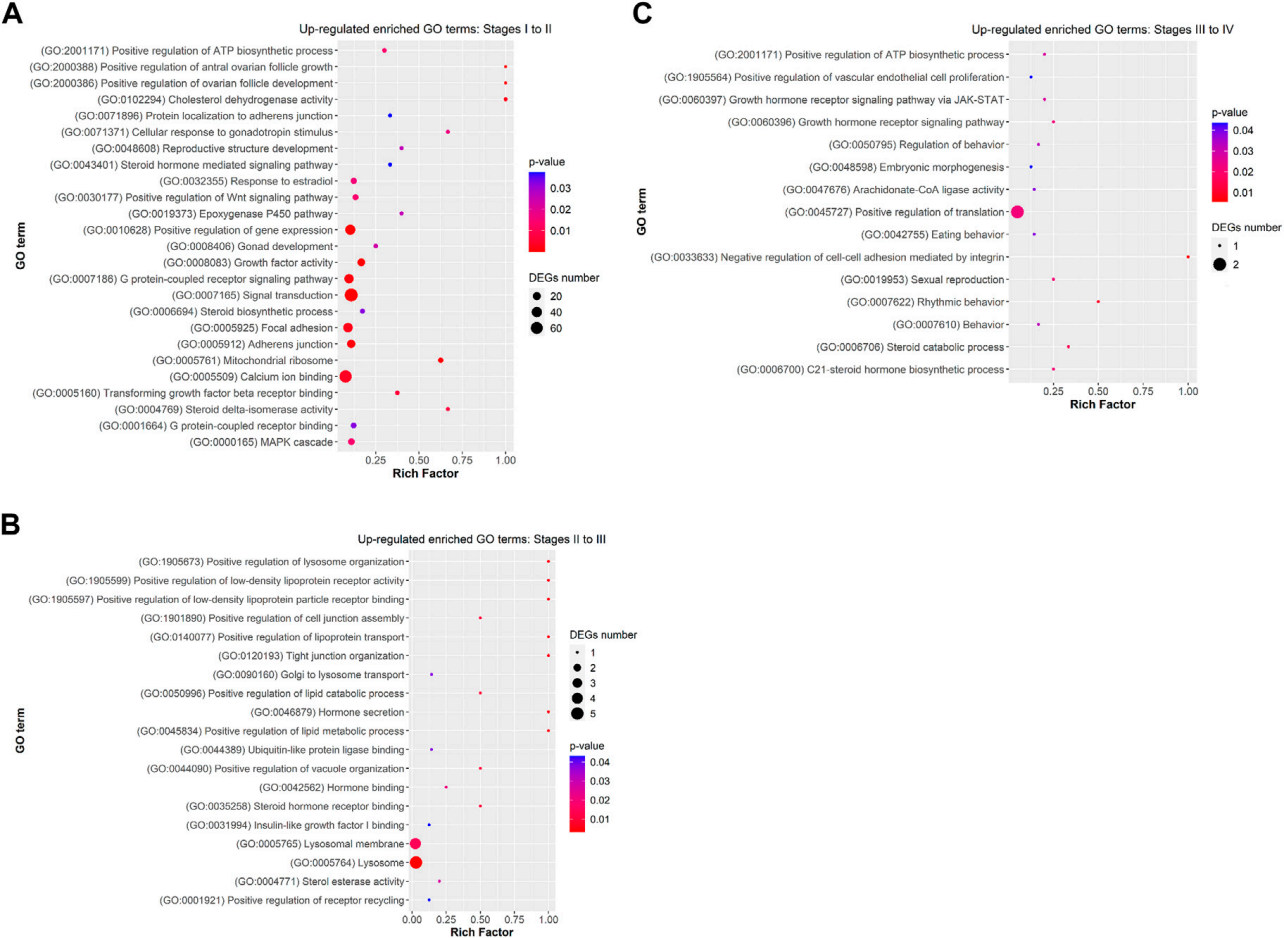
Dot plot showing some relevant significantly enriched Gene Ontology biological processes, cellular components and molecular functions from upregulated DEGs in **(A)** Stage I to II **(B)** Stage II to III, and **(C)** Stage III to IV. Rich factor is the ratio of the differentially expressed gene number to the total gene number in a certain GO term. The colour and size of the dots represent the range of the *p*-value and the number of DEGs mapped to the indicated GO term, respectively.

**FIGURE 6:**
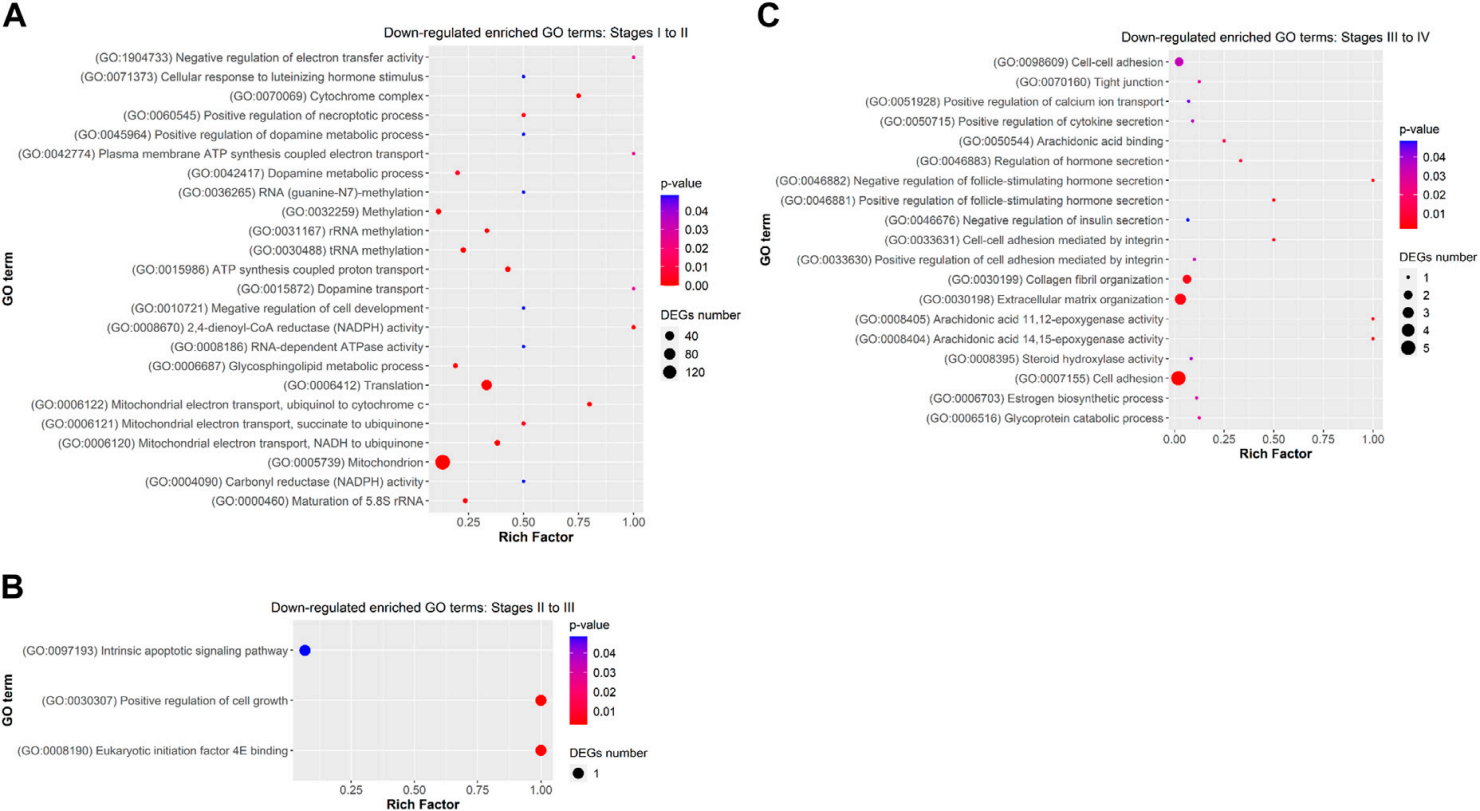
Dot plot showing some relevant significantly enriched Gene Ontology biological processes, cellular components and molecular functions from downregulated DEGs in **(A)** Stage I to II **(B)** Stage II to III, and **(C)** Stage III to IV. Rich factor is the ratio of the differentially expressed gene number to the total gene number in a certain GO term. The colour and size of the dots represent the range of the *p*-value and the number of DEGs mapped to the indicated GO term, respectively.

Functional analysis showed that DEGs from previtellogenesis (Stage I) to early-to-mid-vitellogenesis (Stage II), after rFsh administration, were significantly enriched to 1,890 GO terms and classified into categories of BP with 1,169 GO terms, CC with 307 GO terms, and MF with 413 GO terms. By analysing the significantly enriched terms, we found several closely associated with gonadal development. Some of the significant upregulated DEGs were attached to GO terms (Figure 5A) related to BP that were *cellular response to gonadotropin stimulus (GO:0071371)*, *gonad development (GO:0008406), positive regulation of ovarian follicle development (GO:2000386)*, *response to estradiol (GO:0032355), G-protein-coupled signalling pathway (GO: 0007186)*, *positive regulation of ATP biosynthetic process (GO:2001171)*, and *positive regulation of Wnt signaling pathway (GO:0030177)*. In the CC category, genes were significantly enriched with the terms of *adherens junction (GO:0005912)* and *focal adhesion (GO:0005925)*, and in the category of MF, genes were significantly enriched with the terms of *growth factor activity (GO:0005509)* and *calcium ion binding (GO:0005509),* among others. The terms *gonadotropin secretion (GO:0032274)*, *regulation of follicle-stimulating hormone secretion (GO:0046880)*, *response to follicle-stimulating hormone (GO:0032354)*, *3-beta-hydroxy-delta5-steroid dehydrogenase activity (GO:0003854)*, *lipid binding (GO:0008289)* and *glycerophospholipid biosynthetic process (GO:0046474)* were enriched from upregulated genes but not showing a strong significance (*p* ≥ 0.05). On the contrary, some of the significant enriched downregulated GO terms (Figure 6A) related to BP were *negative regulation of electron transfer activity (GO:1904733)*, *mitochondrial electron transport, ubiquinol to cytochrome c (GO:0006122)* and *glycosphingolipid metabolic process (GO:0006687)*. Downregulated terms related to CC were *cytochrome complex (GO:0070069)* and to MF, *carbonyl reductase (NADPH) activity (GO:0008670)*.

Differentially expressed genes from early-to-mid-vitellogenesis (Stage II) to late-vitellogenesis (Stage III), were significantly enriched to 170 GO terms and classified into categories of BP with 119 GO terms, CC with 17 GO terms, and MF with 34 GO terms. Some of the significant enriched upregulated GO terms (Figure 5B) related to BP were *positive regulation of lipid metabolic process (GO: 0045834)*, *positive regulation of low-density lipoprotein particle receptor binding (GO:1905597)* and *Golgi to lysosome transport (GO:0090160)*. In the CC category were *lysosome (GO:0005764)* and *collagen-containing extracellular matrix (GO:0062023),* and in the MF category: *steroid hormone receptor binding (GO:0035258)* and *Insulin-like growth factor I binding (GO:0031994).*

Differentially expressed genes between late-vitellogenesis (Stage III) and full-grown oocytes (Stage IV), were significantly enriched to 197 GO terms and classified into categories of BP with 125 GO terms, CC with 24 GO terms, and MF with 48 GO terms. Some of the significant upregulated DEGs attached to GO terms (Figure 5C) related to BP were *C-21 steroid hormone biosynthetic process (GO:0006700), rhythmic behaviour (GO:0007622)*, *sexual reproduction (GO:0019953)*, and *embryonic morphogenesis (GO:0048598).* Some of the downregulated enriched GO terms (Figure 6C) related to BP were *positive regulation of cell-cell adhesion (GO:0033630)*, *collagen fibril organization (GO:0030199)* and *estrogen biosynthetic process (GO:0006703),* and to CC, *tight junction (GO:0070160).*

### KEGG pathway enrichment analysis of DEGs

The highest number of significantly enriched KEGG pathways of the differential expressed genes occurred in the transition from Stage I (previtellogenesis) to II (early-to-mid-vitellogenesis), with 105 upregulated DEGs enriched in 17 pathways, and 191 downregulated DEGs enriched in 33 pathways. Among them, *GnRH signalling pathway* was enriched by the higher expression of 6 genes (Supplementary Figure S3A). The enriched downregulated pathways related to ovarian development were *steroid biosynthesis*, *oocyte meiosis* and *progesterone-mediated oocyte maturation* (Supplementary Figure S4A).

The transition from Stage II to III (late-vitellogenesis) was represented by the lowest number of significantly enriched pathways, with only 20 upregulated DEGs annotated in nine pathways. Among them are the pathways of *lysosome*, *various types of N-glycan biosynthesis*, *protein processing in endoplasmic reticulum* and *steroid biosynthesis* (Supplementary Figure S3B).

There were 19 upregulated (Supplementary Figure S3C) and 13 downregulated DEGs annotated in nine and eight significantly enriched pathways, respectively, in the transition from Stage III to IV (full-grown oocytes) (Supplementary Figure S4B). *Steroid biosynthesis*, *tight junction* and *focal adhesion* were significantly downregulated.

## Discussion

Treatment with rFsh and rLh in female flathead grey mullet induced vitellogenesis to the completion of oocyte growth, which finally produced fertile eggs (Ramos-Júdez et al., 2021; Ramos-Júdez et al., 2022). Considering these advances and the potential use of rFsh and rLh in other aquaculture species with similar reproductive disorders or to develop out-of-season breeding programs, in the present study we attempted to elucidate the rGths-induced ovarian development of *M. cephalus* using the bulk RNA-Seq method and *de novo* assembly.

In this study, ovarian biopsies of flathead grey mullet at the gonadal arrested stage and different stages through ovarian-induced development with rFsh and rLh were used for histological section preparations coupled with transcriptome analyses. A total of 287,089 transcripts with an expression value of FPKM ≥1 constituted the final assembly. The average transcript length of the assembled transcripts was 798.43 nucleotides (nt) and comparable to other previously reported assembled fish ovarian *de novo* transcriptomes, i.e., 727 nt (Calduch-Giner et al., 2013). Before running DEG analysis, a PCA was performed and the first two principal components explained 66% of the variance. The first component (PC1) explained 55% of the variance and discriminated among the different stages of oocyte development, while the second component (PC2) explained 11% of the variance. Samples were positioned in the PC1 dimension according to the different stages of ovarian development, which indicated that the variation in the gene expression was associated with ovarian development. Previtellogenic samples that presented some oocytes in the cortical alveolus stage grouped with vitellogenic samples indicating that gene expression changes associated with the cortical alveolus stage were continued into vitellogenesis, which could be related to the fact that these oocytes (cortical alveolus and vitellogenic) belong to the secondary growth stage (Lubzens et al., 2010). There was also an overlap of some molecular signatures between the closest stages of vitellogenesis that could be explained by the type of ovary development this species presents. *Mugil cephalus* females treated with rFsh and rLh treatment (Ramos-Júdez et al., 2021; Ramos-Júdez et al., 2022) and wild females (Kumar et al., 2015) are described to have a group-synchronous ovary in which several populations of oocytes can be distinguished at the same time; a most abundant synchronous clutch of larger oocytes that mature leading to a single annual spawn, and a heterogenous clutch of smaller oocytes from which the clutch is recruited. The overlap may also simply indicate that many of the molecular processes throughout the different stages of vitellogenesis were similar. This partial molecular overlap has also been described in other fish species that have group-synchronous ovarian development, such as the largemouth bass (*Micropterus salmoides*) (Martyniuk et al., 2013). Although this overlap existed, the stages were incremental and characterized by visual changes in the overall appearance of the histological sections. These observations of the PCA oocyte stage grouping and general overview of gene expression were broadly supported by the analysis of DEGs during ovary development that revealed the number of DEGs between Stage I - II, I - III and I - IV were notably greater than those between II - III and III - IV, indicating that many similar genes were expressed in the vitellogenic stages II, III and IV. Furthermore, a large proportion large proportion of DEGs between Stage I - II, Stage I - III, and Stage I - IV were overlapping, suggesting they were explicitly involved in the developmental processes from Stage I. The present study identified a more significant number of significantly upregulated DEGs, with the progression of vitellogenesis, than downregulated ones. Observing an upregulation of genes implies that mRNAs are actively accumulated (Kleppe et al., 2014). A contrary trend for the total number of DEGs between stages of ovarian development has been reported in the largemouth bass (Martyniuk et al., 2013) and in the Atlantic cod (*Gadus morhua*) (Kleppe et al., 2014), where more genes were downregulated than upregulated during gonadal development.

### From previtellogenesis (Stage I) to early-to-mid vitellogenesis (Stage II): Effects of rFsh

The application of rFsh was previously shown to stimulate vitellogenesis in previtellogenic flathead grey mullet females (Ramos-Júdez et al., 2021; Ramos-Júdez et al., 2022) and was further confirmed by the molecular data in the present study that was associated with this process. With the application of rFsh, there was an increase in oocyte size with an accumulation of lipid droplets (Ramos-Júdez et al., 2021 and Ramos-Júdez et al., 2022, present study) and an increase in 17β-estradiol (E_2_) (Ramos-Júdez et al., 2021). This is similar to the described initiation of vitellogenesis when Fsh is understood to act through the follicle-stimulating receptor (*fshr*) in follicle cells and stimulate the synthesis of steroids, among them E_2_ that is released into the bloodstream and in turn stimulates the production of vitellogenin from the liver, which is incorporated into the developing oocyte (Le Men et al., 2007; Lubzens et al., 2010). As expected, a feature from Stage I to Stage II was the transcript upregulation of the *fshr* in response to the exogenous application of rFsh over a period of 28 days. The luteinizing hormone receptor (*lhcgr*) expression levels were low and remained unchanged, which might indicate the specificity of Fsh to express and activate its receptors, as happens in mammals (Tilly et al., 1992) and most of the fish species described such as the rainbow trout (*Oncorhynchus mykiss*) (Sambroni et al., 2007), sea bass (*Dicentrarchus labrax*) (Molés et al., 2011), eel (*Anguilla japonica*) or tilapia (*Oreochromis niloticus*) (Aizen et al., 2012). Probably in response to the *fshr* upregulation, the GO terms of *cellular response to gonadotropin stimulus (GO:0071371)*, *steroid biosynthetic process (GO:0006694)*, *steroid hormone receptor activity (GO:0003707)*, and *positive regulation of ovarian follicle development (GO:2000386)* were enriched from upregulated genes. The enrichment of these pathways would reflect the increase of steroidogenic activity in the ovary for reproductive development. Specifically, at the gene level, genes such as *star, hsd3β*, *cyp17a, cyp19a1,* and *hsd17b* that are involved in steroidogenesis were upregulated in the transition from Stage I to II. For example, *star* mediates cholesterol transfer in the mitochondria (Stocco, 2001) —a rate-limiting step in steroidogenesis (Tiosano et al., 2019)—, *hsd3β* converts pregnenolone into progesterone that together with pregnenolone is converted by *cyp17a* into precursors for the synthesis of androgens and estrogens (Gohin et al., 2010)*,* and *cyp19a1* and *hsd17b* are involved in the conversion of androgens to E_2_ (Kagawa et al., 1982; Uno et al., 2012)*.* Furthermore, the increased expression profile of nuclear estrogen receptors 1 and 2, *esr1* and *esr2* observed may indicate the involvement of estrogens in oocyte development, favouring the change of previtellogenic oocytes from Stage I, from the primary into the secondary oocyte growth phase (García-López et al., 2011).

Given that rFsh stimulates the receptors located in the follicle cells where steroidogenesis takes place (Lubzens et al., 2010), and that the oocyte incorporates molecules containing metabolites, information and nutrients (Rosenfeld et al., 2007; Zhao et al., 2015), it was expected the enriched pathways from upregulated genes to indicate that cell connection occurs and the oocyte communicates with the surrounding follicle cells for the regulation of its growth and development. In this sense, *Focal adhesion* was a GO term and KEGG pathway significantly enriched from Stage I to Stage II. Focal adhesion results in a connection mediated by cells to the extracellular matrix (Zhao et al., 2015). The regulation of *actin cytoskeleton* pathway was also enriched in early-to-mid-vitellogenesis compared to previtellogenesis, a pathway that has an important function in the transport of oocyte-specific RNA (García-López et al., 2011). Besides, *adherens junction* and *tight junction* were both enriched in the transcriptome. Some of the participants of the network that enables this interplay between the follicular cells and the oocyte are the growth and differentiation factors (Rosenfeld et al., 2007). According to this, the expression of ovarian mRNA levels of genes encoding IGF-binding proteins such as *igf2* were found to increase at Stage II, which reflects the possible paracrine involvement of the IGF-system during follicular development in the flathead grey mullet oogenesis under the stimulation by gonadotropins. High ovarian levels of *igf2* during vitellogenesis have been previously described in other fish species (Campbell et al., 2006; García-López et al., 2011; Kleppe et al., 2014). Moreover, the application of rFsh also enhanced the mRNA levels of genes that lead to the enrichment of the *growth factor activity (GO:0008083)* such as the gene encoding for the transforming growth factor beta 1 (*tgfb1*), and other factors, such as bone morphogenic proteins 2, 5 and 7 (*bmp2, bmp5,* and *bmp7*). As previously suggested in other species, in addition to the generally accepted role in ovarian steroidogenesis, Fsh seems to regulate genes associated with ovarian cell-growth, differentiation and survival (Sakai et al., 1994; Kusakabe et al., 2002; Nakamura et al., 2005; Guzmán et al., 2014). The *Wnt signalling pathway* was upregulated and is constituted by glycoproteins that regulate cell differentiation and oocyte survival (Li et al., 2021). The enrichment of these pathways, which has been described in other species’ ovary transcriptomes, is important for the maintenance of the physiological activity of the ovary (Zhao et al., 2015; Jia et al., 2018) and necessary for normal fertility (Bliss et al., 2010). Related to this, the higher expression of forkhead box L2 (*foxl2*) in early-to-mid-vitellogenesis implies that ovary maintenance events were taking place (Jia et al., 2018).

Related to the control of vitellogenesis, it was observed an enrichment from upregulated genes of the *Gonadotropin-releasing hormone (GnRH)* pathway. It is well-known that GnRH secreted from the hypothalamus regulates the production and release of Fsh and Lh. Furthermore, it regulates MAPKs activities, serine/threonine protein kinases that act as a component of signalling transduction in the regulation of cell growth, cell differentiation and cell cycle (Seger and Krebs, 1995). In this sense, *MAPK cascade (GO:0000165)* was also found to be enriched from upregulated genes. Intriguingly, the application of rFsh appears to have a feedback effect on pathways related to the upstream of the brain-pituitary-gonad axis. It has been described that sex steroids produced in gonads, as well as other endocrine signals from peripheral organs, exert a negative and positive feedback effect on GnRH, hence regulating gonadotropin synthesis and secretion (Fontaine et al., 2020). Therefore, the steroids produced under the application of rFsh, might also have a role in a feedback system at the hypothalamic-pituitary level.

The aforementioned Fsh-signaling pathways demonstrated that rFsh stimulated steroidogenesis, and it is well known that the release of E_2_ leads to the hepatic synthesis of vitellogenin, which is incorporated into oocytes to produce egg yolk proteins (Lubzens et al., 2010), together with other molecules such as vitellin and phosvitin (Hara et al., 2016). Therefore, in this respect, in the present study, there was an upregulated enrichment of *glycerophospholipid biosynthetic process (GO:0046474)*, that although not significant, together with a downregulated enrichment of *glycosphingolipid metabolic process (GO:0006687)*, might imply a degradation of lipids for storage (Kleppe et al., 2014). Several genes associated with the production of vitellogenin and the transport or endocytosis of vitellogenin and other very low-density lipoproteins (*lrp1 lrp5, lrp6, apoeb*) were upregulated. The involvement of upregulated *fads6* implied an active lipid metabolism. These findings provide additional data to the reported role of Fsh in the process of yolk uptake in vitellogenesis (Tyler et al., 1991; Kim et al., 2005; Molés et al., 2012; Guzmán et al., 2014). The synthesis and uptake of various substances, i.e., cholesterol or lipids, necessary for the development of the oocyte, are processes of a high energy demand. The binding of rFsh to its receptors might play an important role in the intracellular energy transfer for the several processes induced. For example, the first step in steroidogenesis takes place within mitochondria for which energy is necessary (Rosenfeld et al., 2007). However, a noteworthy aspect of the transition from previtellogenesis to early-to-mid-vitellogenesis was the *cytochrome complex (GO:0070069)* enrichment from downregulated genes. Contrarily to other species, in which mRNA levels of genes involved in mitochondrial respiration and oxidative phosphorylation were upregulated in early-vitellogenesis respect to previtellogenesis (Lokman et al., 2003; Martyniuk et al., 2013; Wylie et al., 2019), in the present study cytochrome *c* (*cycs*) and *b* (*cycb*) transcripts were downregulated. However, a high expression of cytochrome *c* was still present together with an upregulated expression of some *cox* genes, and thus, the proton gradient required for ATP production might have been present. On the other hand, there was a positive enrichment of the *G protein-coupled receptor signalling pathway (GO: 0007186)*, from which the neurotransmitter receptor *npy1r* was identified. The neurotransmitter of neuropeptide Y is described to be an important regulator of energy homeostasis in fish and mammals (Wu et al., 2012), also present in the Atlantic cod (*Gadus morhua*) vitellogenic follicles (Kleppe et al., 2014). This may indicate the activation of processes important for energy uptake and oocyte growth (Kleppe et al., 2014). Taken together, these molecular processes demonstrated how the exogenous administration of just rFsh induced the initiation and progress of vitellogenesis, oocyte growth, and related processes.

### From early-to-mid (Stage II) to late-vitellogenesis (Stage III): Effects of rFsh and rLh

While there was a higher number of DEGs on the transition from Stage I to Stage II, lesser DEGs were observed between Stage II and Stage III, which could be related to the fact that both are close stages from a continuous process; vitellogenesis. The main histological difference that could be observed after the combined application of rFsh and rLh in successive weeks was the growth in diameter of the developing oocytes (from ∼320 to 550 µm in diameter), and oil and yolk droplets. There was little change in the expression levels of the *fshr*, which remained high, or the *lhcgr*, whose expression levels increased according to rLh application at Stage III with respect to previtellogenesis but no significant differences were observed with Stage II. Therefore, the maintained high *fshr* expression appeared to control the steroidogenic activity of the ovary which was further corroborated by the prolonged high E_2_ levels observed in the previous study (Ramos-Júdez et al., 2021). This is perhaps surprising as the combined treatment of rFsh with rLh appeared to be crucial for oocyte growth at 400+ µm (Ramos-Júdez et al., 2021), given that females that only received rFsh exhibited gonadal arrest at this point. The receptor expression profiles suggested the exogenous rLh had a minimal effect on *lhcgr* expression, perhaps giving support to hypothesize that rLh could also activate the *fshr* to further potentiate estrogen production and vitellogenesis. In other fish species, such as in sole (*Solea Senegalensis*) (Chauvigné et al., 2012) and zebrafish (*Danio rerio*) (So et al., 2005), Lh recognizes both receptors, *fshr* and *lhcgr.* However, specific studies on transactivation assays would better confirm the promiscuous Lh-receptors interaction.

Given that similar processes were expected to occur between Stage II and Stage III, i.e., continuous oocyte growth and incorporation of lipids, the joint rFsh and rLh administration maintained significantly enriched pathways from upregulated genes directly related to the reproductive function, steroidogenesis and cell connections, such as *hormone secretion (GO:0046879)*, *steroid hormone receptor binding (GO:0035258)*, *and tight junction organization (GO:0120193).* Other enriched GO terms and KEGG pathways were those related to lysosomes such as *lysosome (GO:0005764)*, enriched by the presence of cathepsins. Lysosomes participate in several cellular processes, i.e., cholesterol homeostasis, cell signalling, macromolecules degradation *etc.* (Carnevali et al., 2006) and might be involved in energy redistribution, which could be related to the production of energy for reproduction, as suggested for the shrimp (*Macrobrachium nipponense*) (Zhang et al., 2021). Therefore, it could be hypothesized that lysosomes played a role in the production of energy during the development of *M. cephalus* vitellogenesis induced by rFsh and rLh.

### From late-vitellogenesis (Stage III) to full-grown oocytes (Stage IV): Effects of rLh

In the transition from Stage III to Stage IV under the stimulation by just rLh, the main morphological difference was that oocytes had completed vitellogenic growth (last ∼50 µm in diameter) and achieved maximum size before the process of oocyte maturation. Both gonadotropin receptors showed similar expression levels within Stage III and IV, but this time expression of *lhcgr* increased, and the termination of rFsh application, caused a decline of *fshr* mRNAs comparing Stage IV with early-to-mid vitellogenesis (Stage II). A similar pattern of receptor expression was previously described in the European sea bass in which *lhcgr* expression increased at advanced vitellogenesis (García-López et al., 2000) and *fshr*, decreased. This suggests that while steroidogenic activity for the completion of vitellogenesis was still taking place, the oocyte was preparing for further processes regulated by Lh and the expression of its receptors. The single application of rLh to complete vitellogenesis immediately before full-grown oocytes may indicate that Lh has a role in vitellogenic growth, generally attributed to Fsh. Therefore, in the flathead grey mullet gonadotropins might not present the dual proposed role as in salmon or mammals (Lubzens et al., 2010; Aizen et al., 2012).

Associated with the preparation of oocytes towards the following steps in oogenesis, which would be oocyte maturation, which includes a shift in steroid production from the synthesis of E_2_ to a C-21 derived steroid, the maturation-inducing steroid 17α, 20β-dihydroxy-4-pregnen-3-one (Lubzens et al., 2010), there was a downregulated enrichment in the *estrogen biosynthetic process (GO:0006703)* and an upregulated enrichment in the *C-21 steroid hormone biosynthetic process (GO:0006700)*. Therefore, the present study adds data to the role of Lh as the driver that triggers this shift in steroid production (Kagawa, 2013). Additionally, concerning oocyte maturation and preparation for spawning, enrichment of pathways from downregulated genes related to cell connections, such as *tight junction*, *focal adhesion*, *actin cytoskeleton,* and *cell-cell adhesion (GO:0007155)* was observed, typical from a late-vitellogenic stage described in the Atlantic cod (Kleppe et al., 2014). In addition, the enrichment of pathways as *embryonic morphogenesis (GO:0048598)* might indicate the preparation of the egg for future development. Surprisingly, paths such as *rhythmic behaviour (GO:0007622)*, *sexual reproduction (GO:001995)* and *behaviour (GO:0007910)* were enriched from upregulated genes, which leads to speculate that the application of rLh promoted the expression of genes that coordinate physiological rhythmic processes, and that are involved in functions relevant to reproductive behaviour.

## Conclusion

In conclusion, RNA sequencing and bioinformatic tools were used to evaluate the ovarian transcriptome development under rGths induction in the flathead grey mullet. The present study described enriched paths with genes being differentially expressed in the ovary as induced vitellogenesis progressed. Regrettably, we cannot conclude whether the described molecular patterns are following those of the flathead grey mullet natural cycle without the use of external recombinant Fsh and Lh. However, the paths and genes described were typical of natural oogenesis in other fish species, and vitellogenesis was proven to be successfully induced with the final production of fertile eggs (Ramos-Júdez et al., 2021; Ramos-Júdez et al., 2022). These data will serve as a baseline for studies that aim to understand the molecular basis of stage-specific physiological events during rGths-induced vitellogenesis in the ovary of teleosts. The description of molecular mechanisms involved in gonadal development under rGths treatment is not only of basic knowledge but also will be of practical relevance for the application of rGths in fish aquaculture breeding programs.

## Data Availability

The datasets presented in this study can be found in online repositories. The names of the repository/repositories and accession number(s) can be found below: https://www.ebi.ac.uk/ena, Project ID: PRJEB53250.
