# Supplementary material for: Transcriptome analysis of flathead grey mullet (Mugil cephalus) ovarian development induced by recombinant gonadotropin hormones
Source: Front Physiol. 2022 Nov 1;13:1033445. doi: 10.3389/fphys.2022.1033445 (PMC9664002; doi:10.3389/fphys.2022.1033445)
Supplement: Supplementary file 1 [file Table1.DOCX]

**Supplementary Figures for**

**Transcriptome analysis of flathead grey mullet (*Mugil cephalus*) ovarian development induced by recombinant gonadotropin hormones**

Sandra Ramos-Júdez, Theodoros Danis, Nelina Angelova, Alexandros Tsakogiannis, Ignacio Giménez, Costas S. Tsigenopoulos, Neil Duncan and Tereza Manousaki

Sandra Ramos-Júdez, E-mail: [sandra.ramos@s2aquacolab.pt](mailto:sandra.ramos@s2aquacolab.pt)

Tereza Manousaki, E-mail: tereza@hcmr.gr

**This PDF file includes:** Figs. S1 to S4

**Figure S1.** Number of up- and downregulated differentially expressed genes (DEGs) between different sampling points that show different stages of oogenesis induced by recombinant gonadotropins hormones, recombinant follicle-stimulating (rFsh) and luteinizing hormones (rLh), in flathead grey mullet. Stage I, previtellogenesis; Stage II, early-to-mid-vitellogenesis induced with rFsh; Stage III, late-vitellogenesis after combined treatment with rFsh and rLh; Stage IV, full-grown oocytes after rLh treatment.


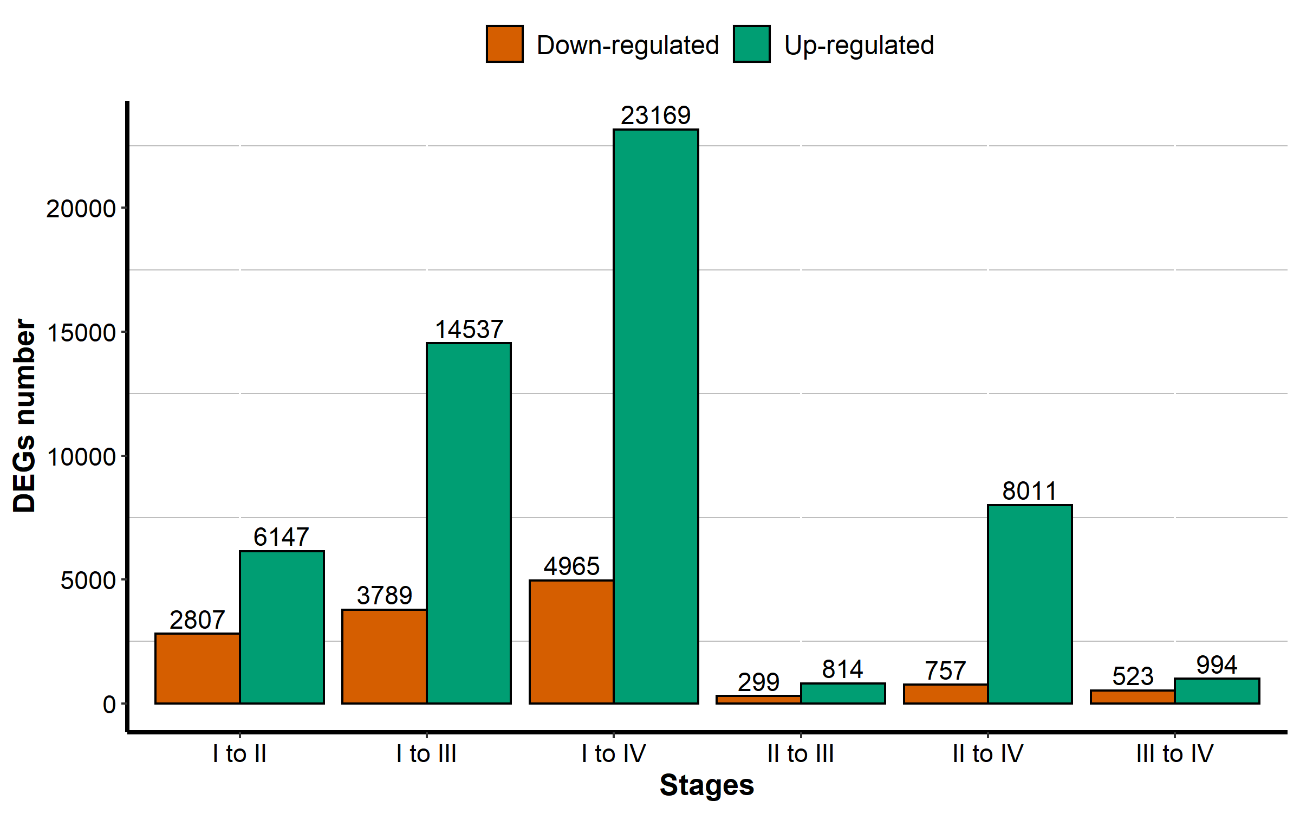


**Figure S2.** Venn diagrams of up/down-regulated DEGs for (**A**, **B**) the comparisons among Stage I vs II, II vs III, and III vs IV, and (**C**, **D**) for the comparisons between Stage I vs II, III and IV.


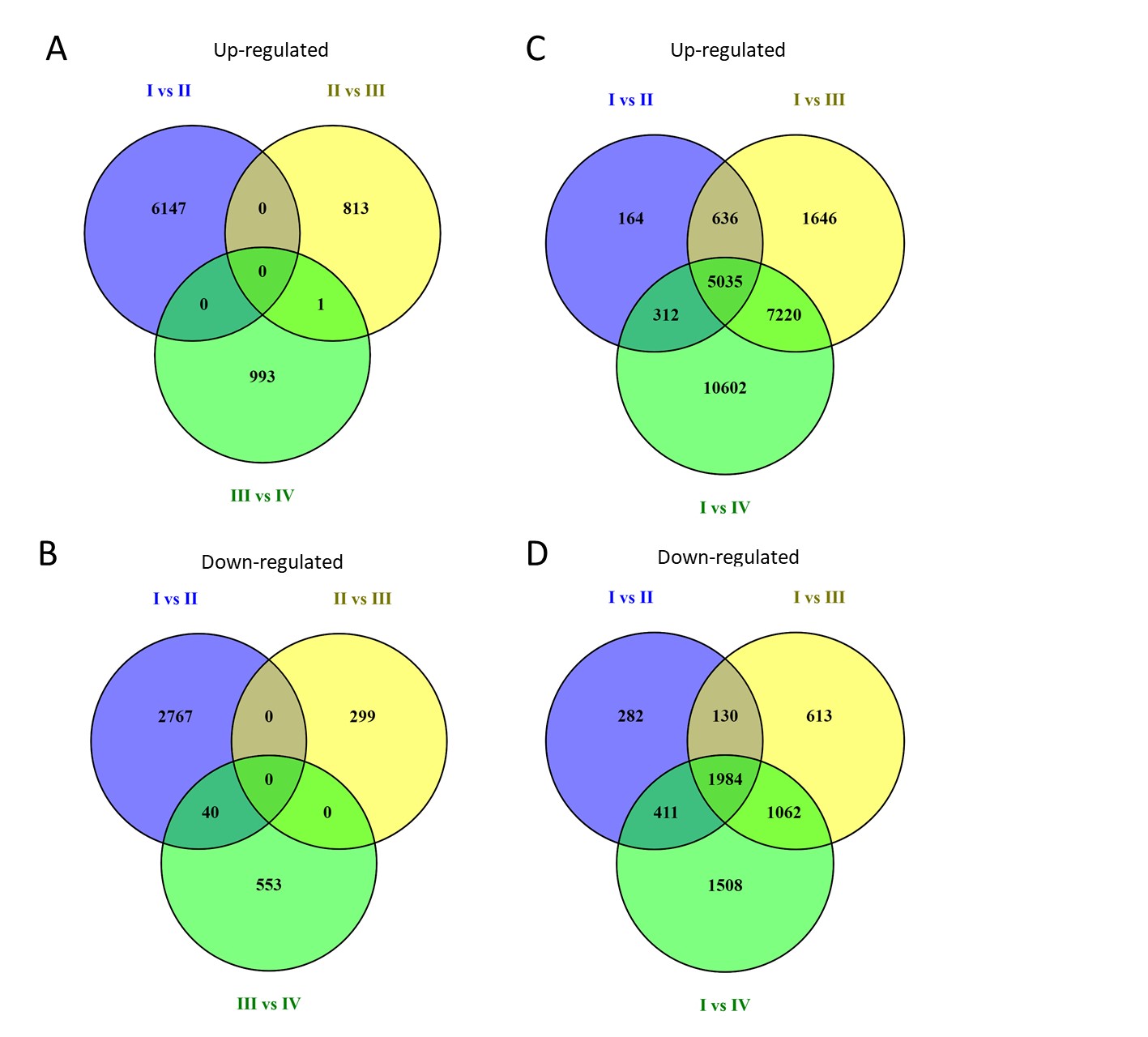


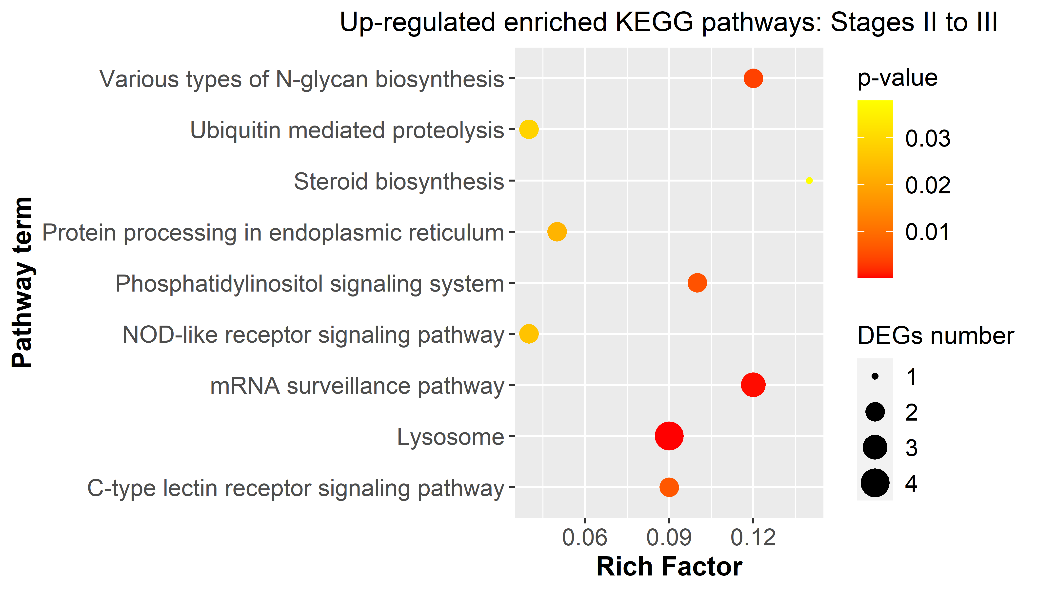
**Figure S3.** KEGG pathway enrichment analysis of up-regulated differently expressed genes (DEGs) between (**A**) Stages I and II (top 20 terms), (**B**) II and III, and (**C**) III and IV. Rich factor is the ratio of the DEG number to the total gene number in a certain pathway. The color and size of the dots represent the range of the *p*-value and the number of DEGs mapped to the indicated pathways, respectively.

**B**


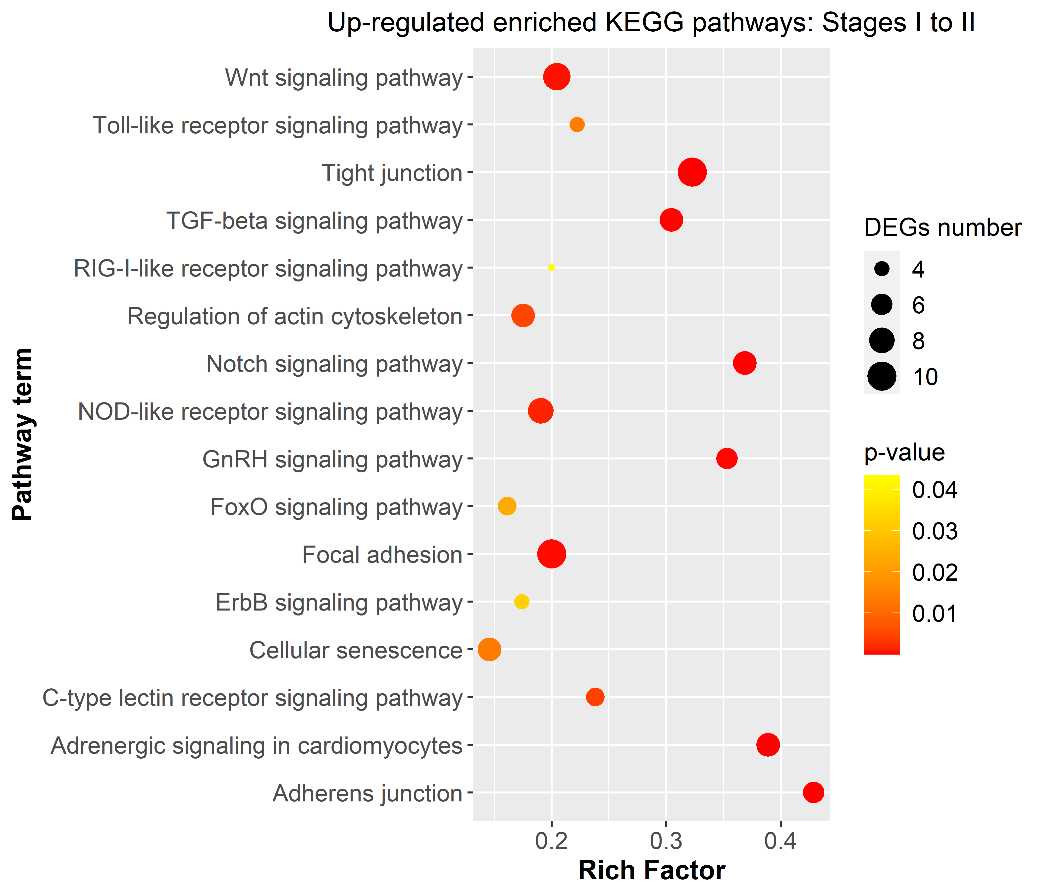
**A**


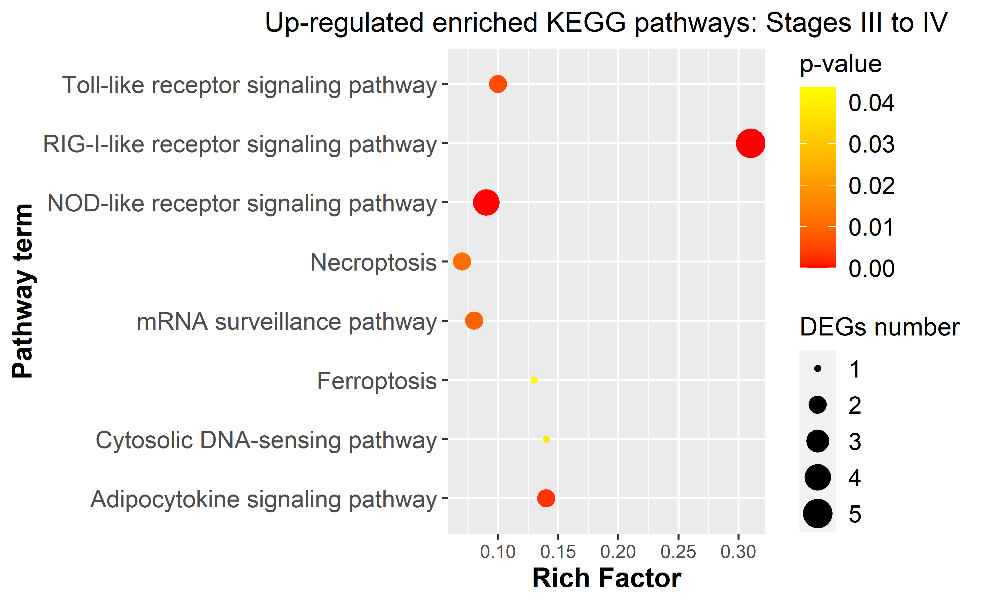


**C**

**Figure S4.** KEGG pathway enrichment analysis of down-regulated differently expressed genes (DEGs) between (**A**) Stages I and II (top 20 terms), and (**B**) Stages III and IV. Rich factor is the ratio of the DEG number to the total gene number in a certain pathway. The color and size of the dots represent the range of the *p*-value and the number of DEGs mapped to the indicated pathways, respectively.

**B**


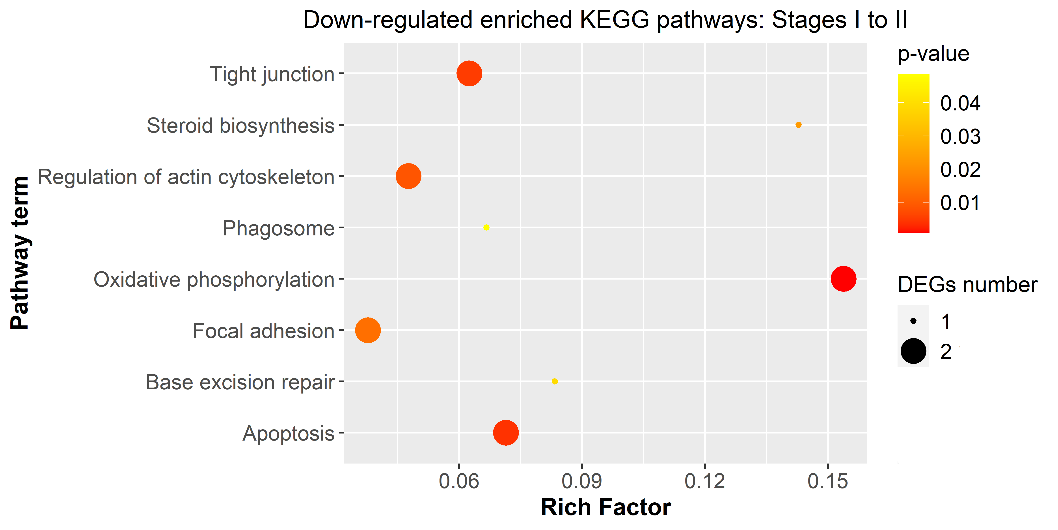

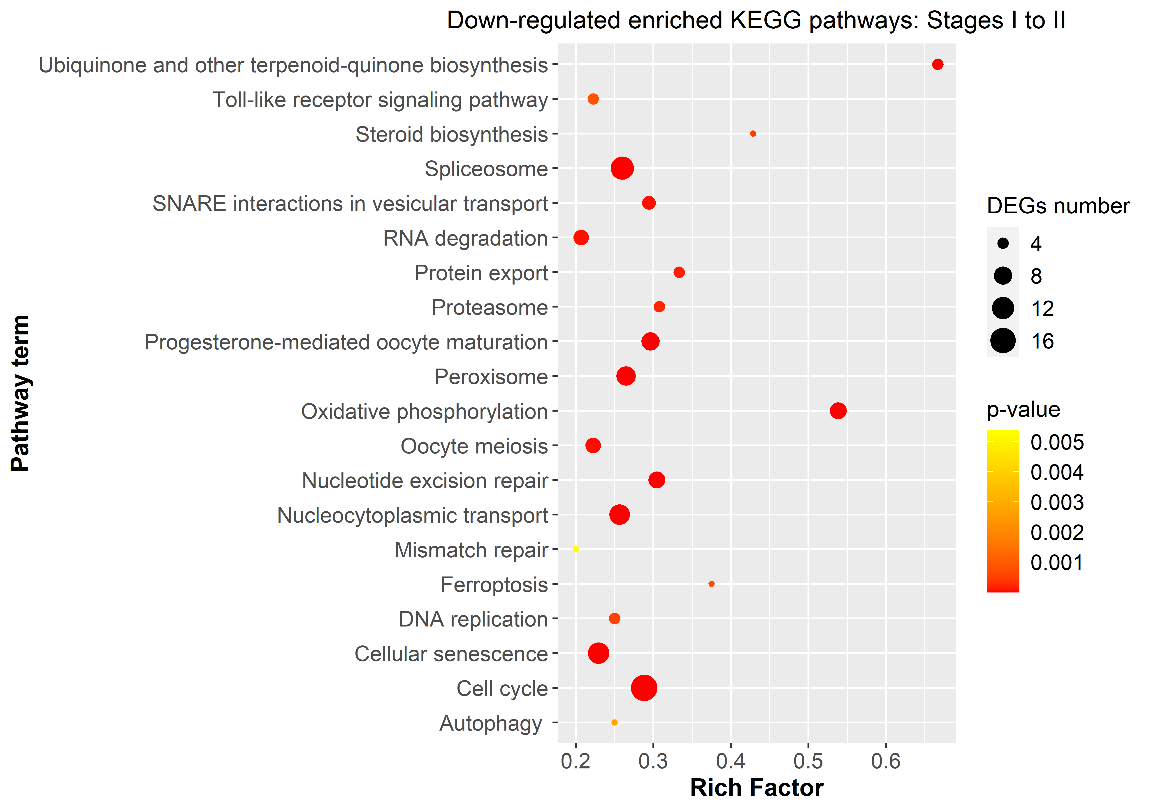
**A**
